# Evolution of the graphite surface in phosphoric acid: an AFM and Raman study

**DOI:** 10.3762/bjnano.7.180

**Published:** 2016-11-30

**Authors:** Rossella Yivlialin, Luigi Brambilla, Gianlorenzo Bussetti, Matteo Tommasini, Andrea Li Bassi, Carlo Spartaco Casari, Matteo Passoni, Franco Ciccacci, Lamberto Duò, Chiara Castiglioni

**Affiliations:** 1 Department of Physics, Politecnico di Milano, p.za Leonardo da Vinci 32, I-20133 Milano, Italy; 2 Department of Chemistry, Materials and Chemical Engineering "Giulio Natta", Politecnico di Milano, p.za Leonardo da Vinci 32, I-20133 Milano, Italy; 3 Department of Energy, Politecnico di Milano, via Ponzio 34/3, I-20133 Milano, Italy

**Keywords:** atomic force microscopy (AFM), electrochemical atomic force microscopy (EC-AFM), electrochemical delamination of graphite, graphene, phosphoric acid, Raman spectroscopy

## Abstract

Phosphoric acid is an inorganic acid used for producing graphene sheets by delaminating graphite in (electro-)chemical baths. The observed phenomenology during the electrochemical treatment in phosphoric acid solution is partially different from other acidic solutions, such as sulfuric and perchloric acid solutions, where the graphite surface mainly forms blisters. In fact, the graphite surface is covered by a thin layer of modified (oxidized) material that can be observed when an electrochemical potential is swept in the anodic current regime. We characterize this particular surface evolution by means of a combined electrochemical, atomic force microscopy and Raman spectroscopy investigation.

## Introduction

Sulfuric (H_2_SO_4_), perchloric (HClO_4_) and phosphoric (H_3_PO_4_) acid in aqueous solutions have been used traditionally for the intercalation of anions in graphite in order to produce graphene [1]. At a given electrochemical potential, suitably defined for a given acid, the layer–layer interaction in the graphene crystal is reduced, facilitating a delamination. In general, after the electrochemical (EC) treatment, graphite is carefully ultrasonicated to ease the exfoliation process. After that single- or multi-layer graphene sheets with a size of about 1 μm can be retrieved from the electrochemical bath. The electronic and mechanical properties of the graphene sheets [1–6] and the main characteristics of the graphite crystals subjected to EC delamination [6–7] have been studied extensively. This allows one to shed light on the correlation between the modifications induced on the graphite crystal and the structure of the exfoliated graphene sheets. H_2_SO_4_ produces blisters at the micrometer scale, i.e., local swellings of the surface caused by the production of gases (O_2_, CO and/or CO_2_) due to the oxidation processes occurring at high anodic potentials [7]. At the nanometer scale, protrusions have been observed together with an increase of the surface roughness caused by graphite oxidation [7–8]. HClO_4_ solutions show a similar phenomenology during intercalation of anions [7]. In particular, the evolution of blisters as a function of time has been analyzed in the past [9–10], supporting the theoretical model proposed by Murray [11]. H_3_PO_4_ is another solvent that allows for successful graphite exfoliation, as reported quite recently [12–13]. However, a detailed analysis of the surface modification of a graphite crystal subjected to EC processes in phosphoric acid solution is still missing. In a recent work [7], we have shown that the EC characterization of the system, i.e., the cyclic-voltammetry (CV) curve, presents a single feature during the first EC potential sweep, but it disappears during the second scan. On the other hand, EC atomic force microscopy (EC-AFM) measurements, performed in situ in the EC cell, reveal a significant increase of the surface roughness. This result suggests that, despite of the good graphite delamination yield, the microscopic processes occurring at the solid–liquid interface could be different from those described in the case of H_2_SO_4_ and HClO_4_ solutions.

In this paper, we focus our investigation on the processes occurring at the graphite surface during EC treatment in H_3_PO_4_, by using both (EC-)AFM (ex situ and in situ) and Raman spectroscopy (ex situ). A correlation between the observed morphology and spectroscopic properties of the surface helps to clarify the effects of phosphoric acid on graphite.

## Results and Discussion

**Figure 1:**
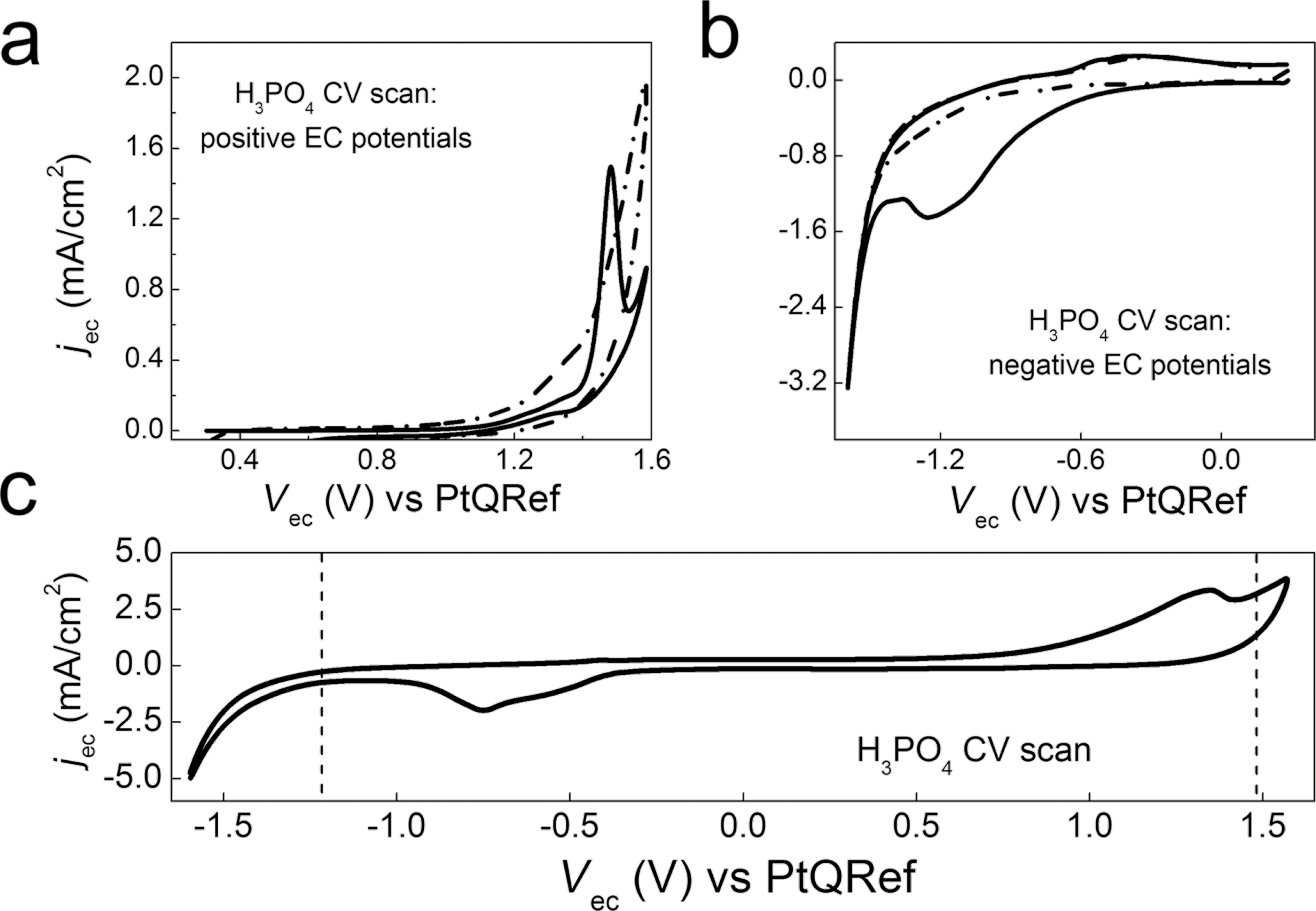
CV in H_3_PO_4_ at different EC potential ranges (scan rate = 150 mV/s). a) First (continuous line) and second (dash-dotted line) scan from 0.3 V to 1.6 V. b) First (continuous line) and second (dash-dotted line) scan from 0.3 V to −1.6 V. c) extended CV from −1.6 V to 1.6 V; the dashed lines mark the original position of the anodic and cathodic peaks.

Graphite acts as working electrode (WE) in the EC cell (see Experimental section for further information). The EC potential is initially fixed at about +0.3 V with respect to the Pt reference electrode (RE). At this potential, the current flowing through the WE is negligible and EC processes do not occur. When the EC potential is swept towards more positive values, the anodic current increases and a clear feature in the cyclic voltammetry (CV) curve is observed at about +1.48 V (Figure 1a), indicating that a charge transfer process is activated at the graphite electrode.

Interestingly, no CV peaks are observed during the second anodic sweep, despite the fact that an enhancement of the oxidation current is always measured. A similar behavior is observed when the CV is confined in the negative EC potential range (see Figure 1b). If the CV is extended from negative to positive EC potentials, both the anodic (in the positive potential range) and cathodic (in the negative potential range) peaks are visible, suggesting that these two EC processes are coupled. The positions of the anodic (at 1.48 V in panel a) and cathodic peak (at −1.25 V in panel b) change when the CV is acquired on the whole range from negative to positive EC potentials. In this case, see panel c, the anodic feature is placed at about 1.40 V, while the cathodic peak is at −0.75 V suggesting that the processes require a lower activation energy.

**Figure 2:**
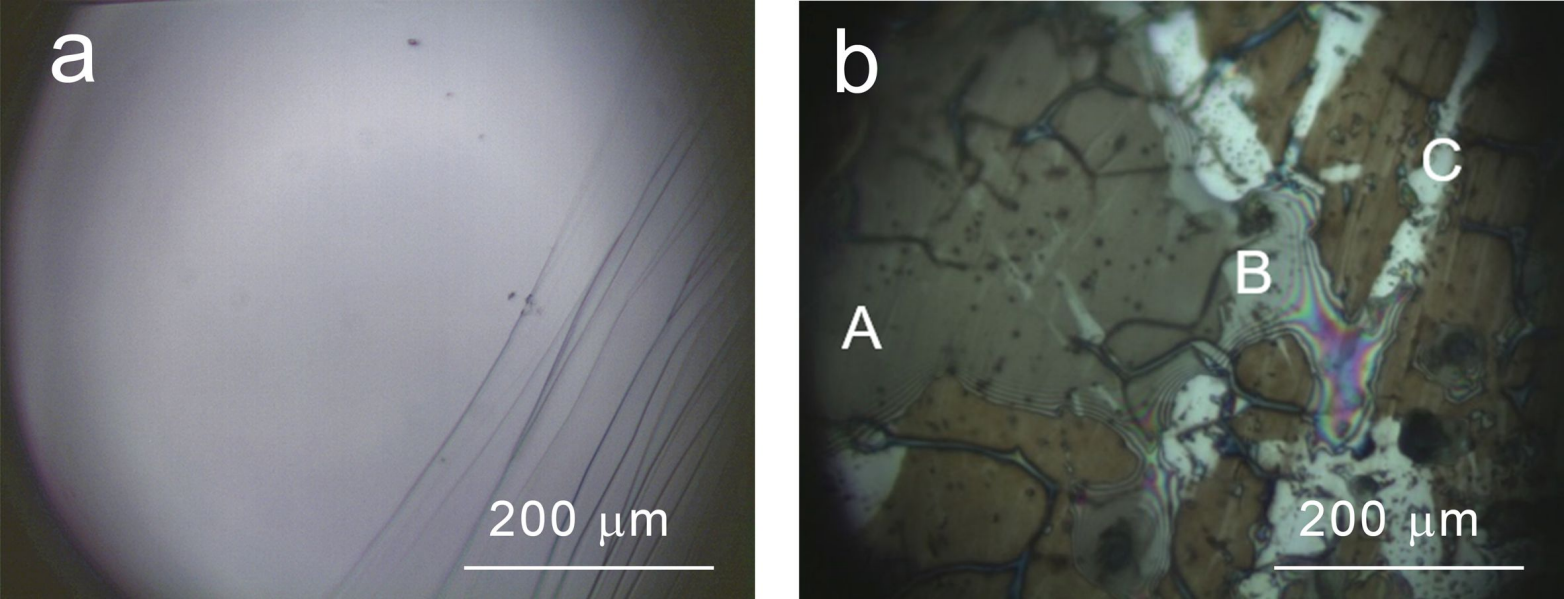
Optical microscopy image (magnification 50×) acquired ex situ on a) pristine graphite and b) graphite after 15 CVs from 0.3 to 1.6 V. Three different areas are observed: a) a thick brown film is recognized, b) a thinner region of the film where interference fringes are clearly visible, C) areas where no modifications can be observed.

Aiming at a first investigation of the sample by optical microscopy, we confined the CV in the positive EC energy range (from 0.3 V to 1.6 V), where oxidation phenomena occur (in close comparison with the case of sulfuric and perchloric solutions [7]). We also cycled graphite fifteen times to enhance the peculiar surface changes induced by the phosphoric acid solution. Optical microscopy reveals an irregular surface, where three main regions can be identified (Figure 2). The majority of the surface is covered by an apparently thick brown film (label A in Figure 2).

Between these A-areas, we observe characteristic interference fringes (B-regions), which suggests that a thinner film is grown there with respect to the darker A-regions. Considering that (1) the EC process induces an oxidation of the graphite surface (anodic currents), (2) the refractive index of graphene oxide is about 1.85 [14–15] and that (3) the light used to acquire the image reported in Figure 2 is in the visible range, it is possible to roughly estimate that the film thickness in the B-areas is of the order of hundreds of nanometers. From this initial optical analysis of the sample, areas labeled as C seem not to be affected by any surface modification.

**Figure 3:**
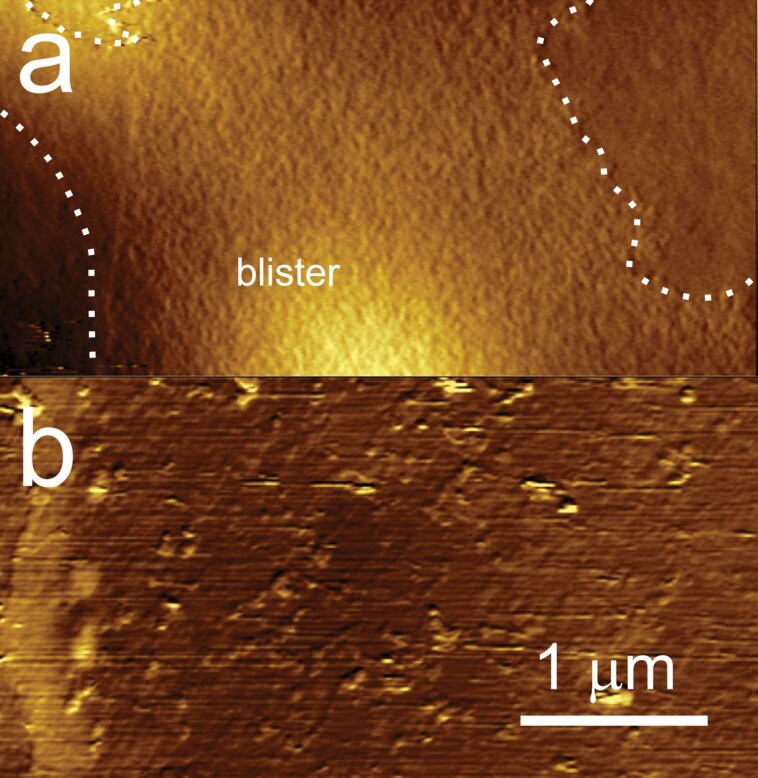
AFM topography images of the HOPG surface after the activation of the electrochemical process in H_3_PO_4_ described in the text. a) Characteristic topography image acquired over the A-region (see text for details), after positioning the AFM tip with the help of a CCD camera. The dotted line highlights the area covered by a rough film. At the bottom of the image, part of a blister is visible; b) characteristic topography image acquired over the C-region (see text for details).

A deeper morphological analysis has been conducted on the same regions by ex situ AFM experiments. Figure 3a shows that the A-area, where the modified film is unquestionably present, is characterized by a high surface roughness (*R*_q_ = 0.3 nm from AFM analysis, which is about five times higher than characteristic mean square root values measured on pristine graphite), conversely to what is observed when sulfuric and perchloric acids are used in the electrolytic solutions. In the latter cases, (nano-)protrusion and blisters characterize the graphite surface after the EC intercalation [7].

Nevertheless, some blisters, randomly distributed on the surface, also occur after the treatment with H_3_PO_4_ acid, as shown in Figure 3a. No differences are observed between A- and B-areas by AFM. C-areas (see panel b) seem to be not affected by the presence of a surface film, but the surface is far from being clean, appearing seriously damaged and dusty.

**Figure 4:**
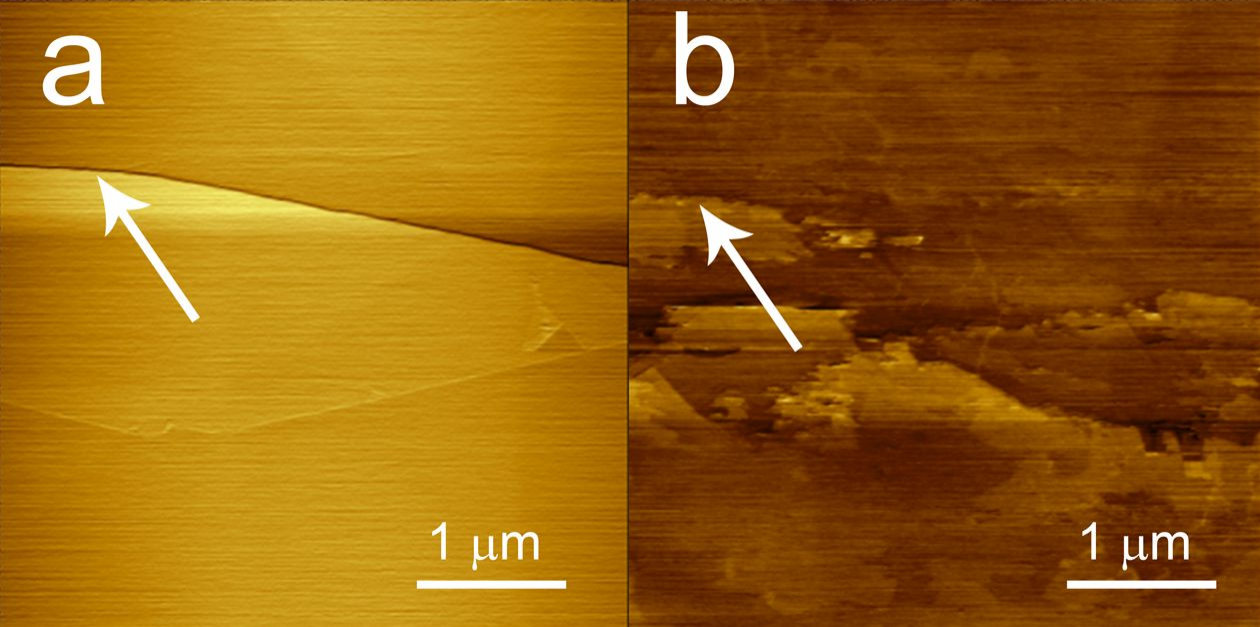
AFM topography images of the HOPG surface a) before EC treatment in phosphoric acid and b) after a single EC potential sweep in the positive energy range. The surface is damaged and eroded because of the EC process. The arrows help to recognize the original graphite step.

To get further insight, we succeeded in following in situ the graphite degradation in these regions during the EC treatment by means of an EC-AFM. Figure 4 compares the surface topography before (panel a) and after (panel b) a single EC potential sweep in the positive potential range. The graphite steps are eroded during the EC process as well as the terraces, suggesting the occurrence of graphite dissolution at these high EC potentials.

**Figure 5:**
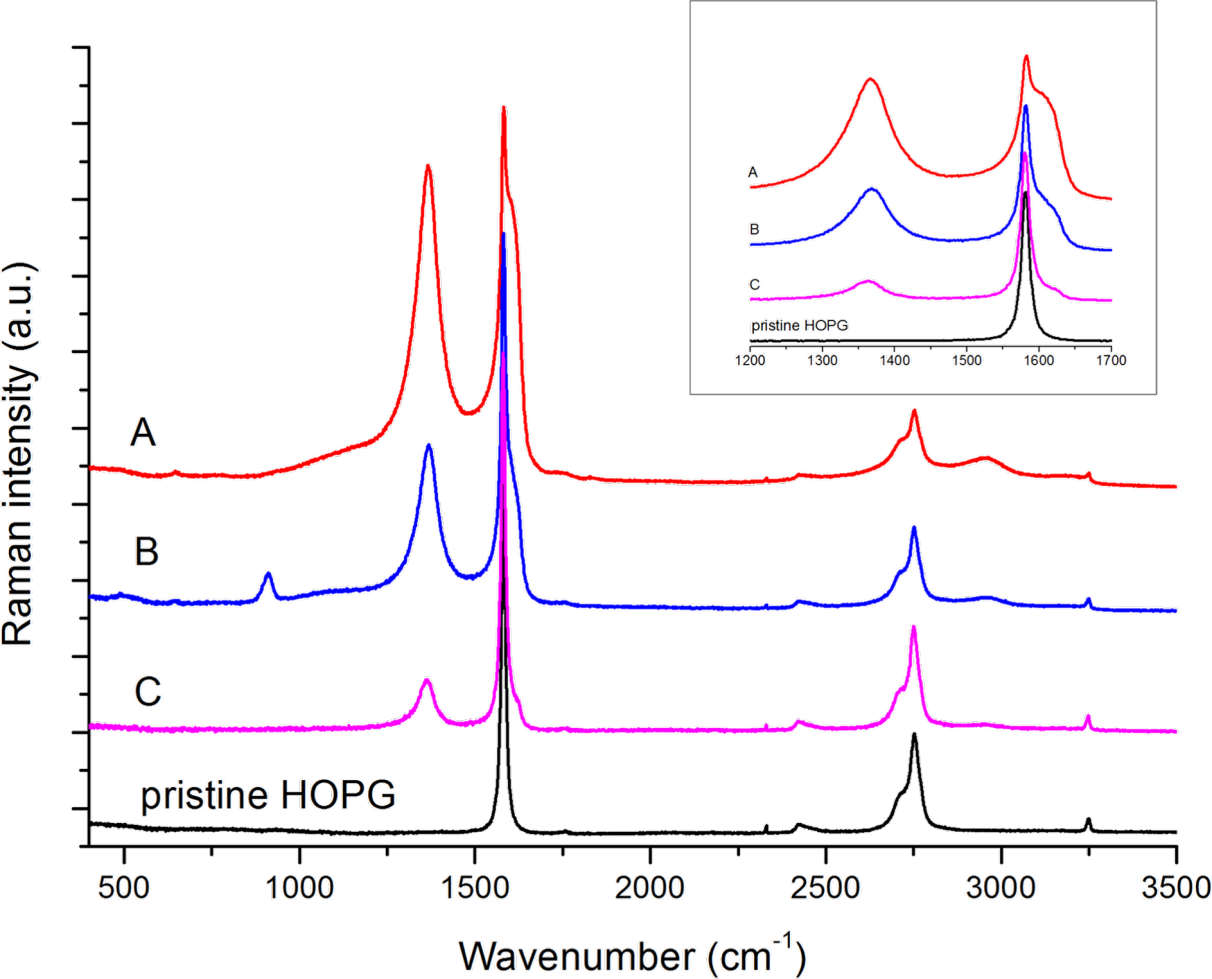
Raman spectra (excitation wavelength of 457.9 nm) of the HOPG sample subjected to 15 CV cycles in the range from +0.3 V to +1.6 V (see Figure 1a). From the top: spectrum of region A (red line); region B (blue line); region C (magenta line). The Raman spectrum drawn with the black line has been taken from a region of the sample not affected by the EC process and it can be considered as a representative reference spectrum of the pristine material (HOPG).

Turning back to the A*-* and B*-*areas, a chemical/structural analysis of the film surface requires a spectroscopic characterization carried out by Raman. Hence, we recorded ex situ several micro-Raman spectra with different excitation wavelengths to get information about the different regions of the sample. We analyzed the HOPG sample after fifteen CV cycles in the positive potentials range (see Figure 1a), focusing the 457.9 nm laser at the A, B and C regions. The four spectra compared in Figure 5 are representative of the pristine HOPG and of the A-, B-, C-regions displayed in Figure 2.

Similar observations can be made, independently on the excitation energy adopted. Indeed, spectra recorded with 632.8 nm and 784.5 nm excitations show a qualitative behavior similar to that observed with 457.9 nm excitation and are not reported in Figure 5. The three different analyzed regions display the typical spectral pattern of graphitic materials characterized by structural disorder [16–18], showing Raman features in the G-band region (1570–1650 cm^−1^), in the D-band region (1250–1400 cm^−1^) and several features belonging to the second-order Raman spectrum. However, the intensity pattern and/or band shapes are different in the three regions analyzed, in particular:

- In the spectra of the A*-*region the D band dominates and, close to the sharp G line at 1582 cm^−1^ typical of HOPG, a rather strong and broad new component appears (hereafter referred as G* band) at higher Raman shifts.
- Similar features can be observed in the B-region. However, in this case both the D line and the G* components are less pronounced than in the A-region. Moreover, an additional new line is observed at 913 cm^−1^. In analogy to the experimental findings reported in [19], this line may be tentatively assigned to Raman modes characteristic of a hydrogen bonded network involving phosphoric acid, its anions and water molecules adsorbed on the graphite surface.
- The C-region, which displays an optically smooth surface, has a remarkably different spectrum, with a strong G line and weak D and G* bands.

**Figure 6:**
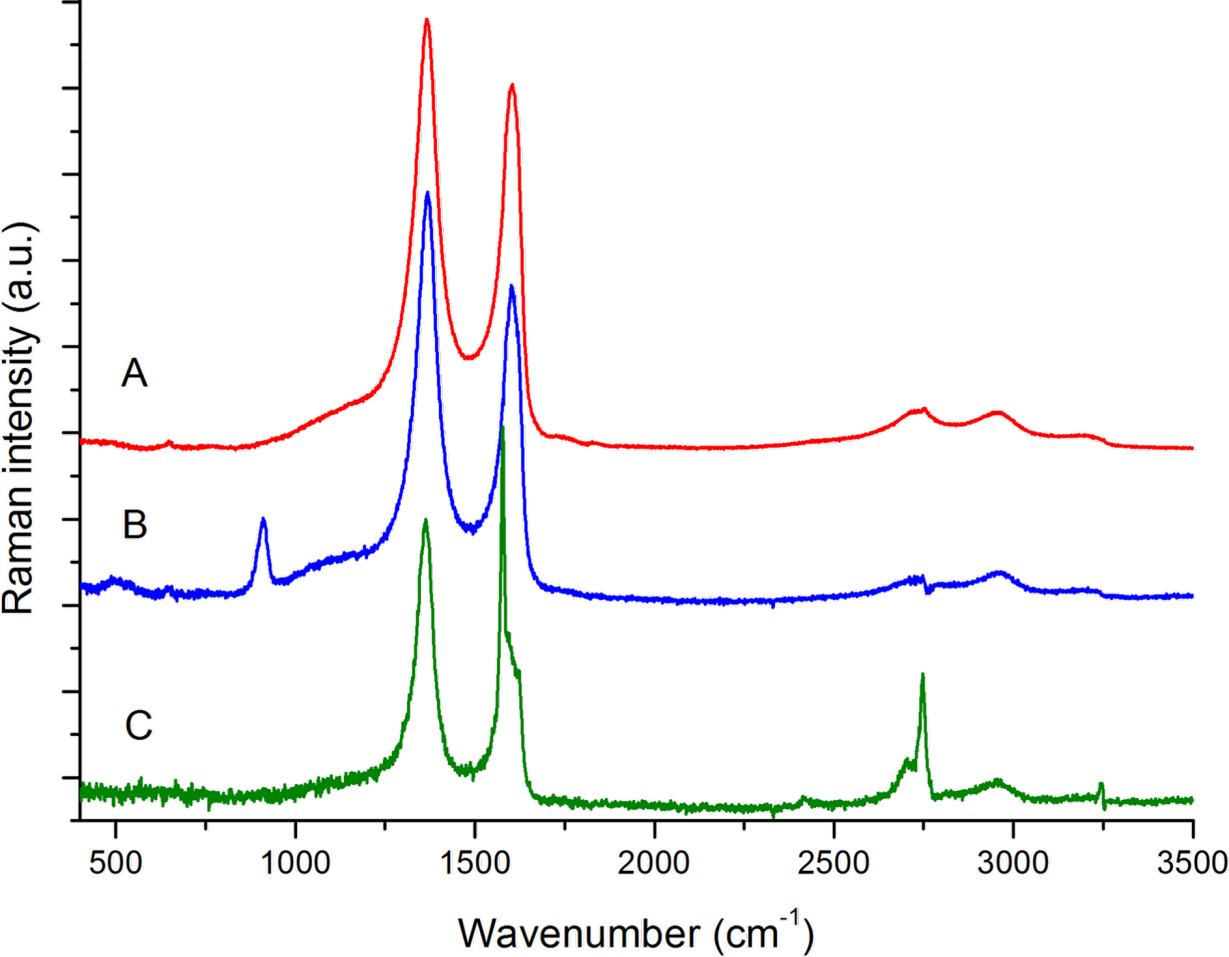
Spectral subtraction of the spectrum of the pristine HOPG from the Raman spectra of: A-region (red line), B*-*region (blue line) and C-region (green line) (see also Figure 5). Excitation at 457.9 nm.

The above observations clearly indicate that the signals observed in the spectra originate: i) from a complex disordered material made by several components, differently modified by the EC process and ii) from coexisting pristine HOPG. Hence, the results from Raman experiments can be better discussed by subtracting the spectrum of pristine HOPG from the spectra of the A-, B-, and C-regions (Figure 6). This was done by means of the standard procedure for spectral subtraction. The difference spectra have been obtained by suitably weighting the spectrum of the pristine HOPG, in such a way that the sharp G line assigned to the HOPG phase disappears (optimal compensation).

The difference Raman spectra of regions A and B (Figure 6) are characterized by a very strong D feature at 1366 cm^−1^ and by the broad G* feature with a maximum at 1604 cm^−1^. This is blue-shifted by 22 cm^−1^ with respect to the G line of HOPG. Moreover, also the sharp 2D feature at 2750 cm^−1^ typical of HOPG turns out to be fully compensated by the subtraction procedure. However, after subtraction we still observe other second-order features, which are commonly found in disordered carbon materials or graphene molecules [20]. The Raman features of the disordered phase show remarkable analogies to the Raman spectra of samples of graphene oxides subjected to chemical reduction [21]. This analogy suggests that the material formed at the graphite surface is the result of the EC oxidation of graphene sheets, possibly followed by partial reduction. Interestingly, the difference Raman spectrum of the B-region is practically superimposable to that of the A-region, with the only exception of the sharp feature at 913 cm^−1^.

**Figure 7:**
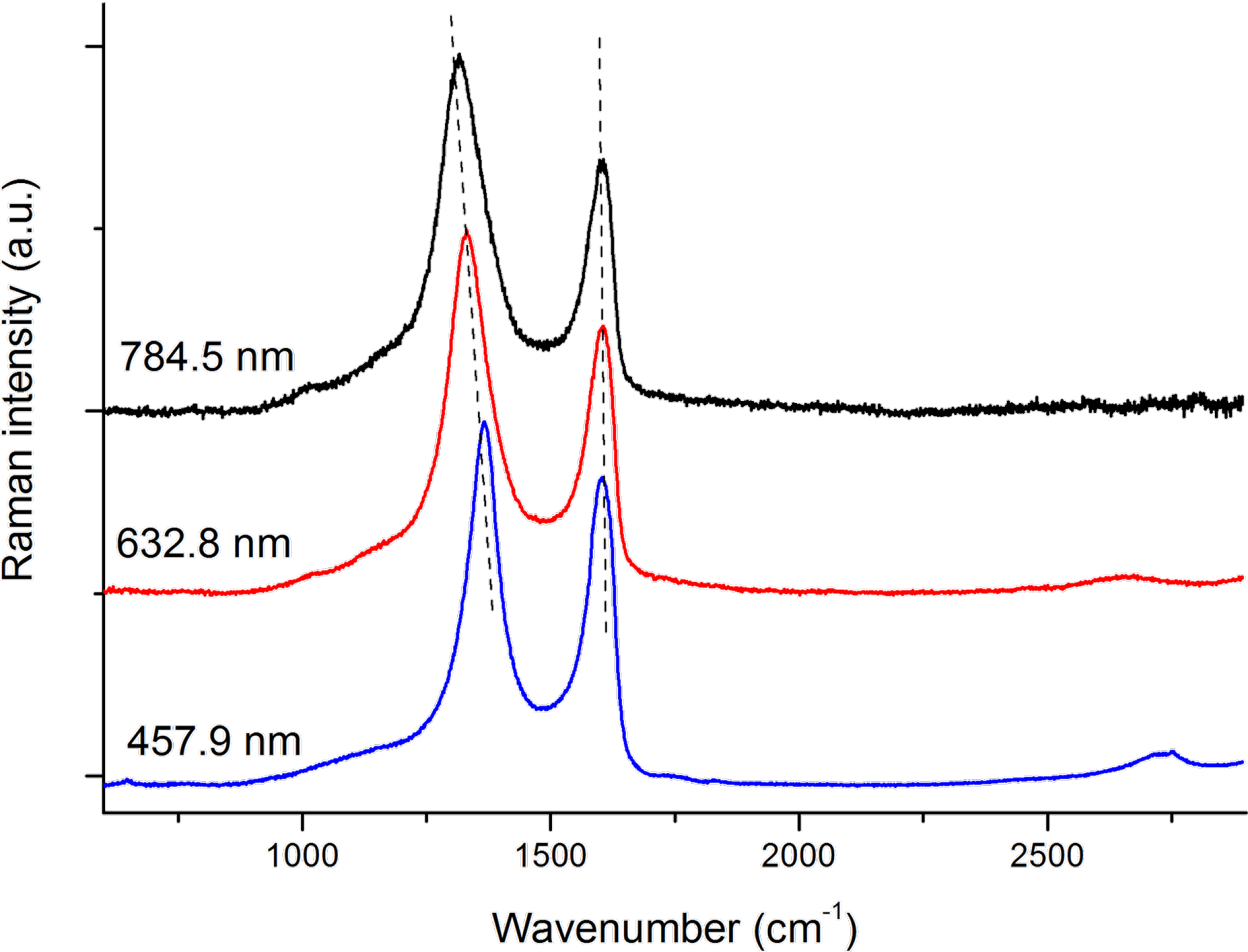
Spectral subtraction of the spectrum of the pristine graphite from the Raman spectra of the A region. From top to bottom: Raman excitation at 784.5 nm (black line), 632.8 nm (red line) and 457.9 nm (blue line). The D peak shows a dispersion of 44 cm^−1^/eV, similar to that observed in microcrystalline graphite [19,22].

We report in Figure 7 the difference Raman spectra of the A-region obtained with the same procedure described above but at different excitation wavelengths. It is evident that modified graphitic species contribute to the spectra of Figure 7, independently of the chosen excitation wavelength. The position of the D line progressively red-shifts with increasing the laser wavelength, as usual in graphitic materials [16,22]. The observed behavior is often interpreted in the framework of the double resonance theory [23], firstly developed for the interpretation of the frequency dispersion observed in multi-wavelength Raman spectra of microcrystalline graphite [22]. However, the sample under investigation contains chemically modified material, probably consisting in a mixture of different, variously defected sp^2^-hybridized layers. In this case the observed frequency dispersion of the D line should be rationalized as the consequence of the Raman response of graphitic domains of different size/perfection, which is selectively intensified by resonance effects [18,24–25].

We may thus conclude that the A- and B-regions consist of a strongly modified graphitic component, which forms the highly rough surface measured with AFM. This film is grown on the HOPG surface, which is simultaneously probed by Raman spectroscopy. Notice that the difference spectra reported in Figure 6 are normalized, while comparing A with B in Figure 5 shows that the contribution of the disordered phase is lower in B. The last observation supports the idea that the modified film is thinner in the B-region, also in agreement with the interference fringes observed in optical microscopy (Figure 2).

A completely different result is obtained by subtracting the reference pristine HOPG spectrum from the Raman spectrum recorded over the optically smooth C-region (Figure 6). In this case a very sharp component of the G line persists, close to the characteristic line of HOPG, but red-shifted by 4 cm^−1^. On the high frequency wing of this G line a rather complex G* feature appears, which does not correspond to the G* band observed over the regions A and B. This feature could be ascribed to some ion intercalation process, by analogy with Raman spectra reported in the literature [26–27]. However, HOPG samples affected by ions intercalation usually show very sharp characteristic lines in the G band region, whose peak position can be correlated to the intercalation stage [26–27], while in this case we observe a broad and structured feature with a peak at about 1622 cm^−1^. Alternatively, we may interpret this spectral feature as partially exfoliated graphene sheets, characterized by structural defects as edges and/or holes. These two hypotheses should be carefully validated by further experiments carried out in different CV regimes and cycling.

## Conclusion

The effect of phosphoric acid solution (2 M) on the surface of HOPG submerged in an EC bath has been studied by conventional cyclic voltammetry (CV). The EC characterization reveals the presence of both anodic and cathodic peaks at 1.48 V and −1.25 V, respectively. The crystal oxidation process, generally exploited for graphite delamination, occurs in the positive potential range (anodic). Optical microscopy investigation reveals the presence of a thick layer deposited on the surface. The topographic analysis of this film has been performed ex situ by means of AFM, while the changes induced by phosphoric acid on the surface of graphite, during the very first stages of the EC treatment, have been monitored in situ by an EC-AFM, which reveals a clear erosion of the surface as a consequence of the high anodic potentials used in the cycle.

A detailed ex situ Raman analysis with three different excitation energies has been carried out on a HOPG sample after fifteen CV cycles in the anodic region. The Raman results corroborate the conclusions derived from the inspection of the sample with optical microscopy and AFM topography. A- and B-regions consist of pristine HOPG material co-existing with a surface layer of highly modified and disordered graphitic phase. The Raman spectra suggest that the disordered component is less abundant over the B-region, where optical microscopy indicates a thinner modified surface layer. The C-region is compatible with incipient anion intercalation and/or exfoliation of small graphene sheets. However, a wider EC-AFM/Raman analysis on samples subjected to different CV treatments is required in order to definitely assess the ability of phosphoric acid to give HOPG intercalation, especially in relation with the exfoliation mechanism.

## Experimental

### Sample and electrochemistry

As working electrode (WE) Z-grade highly oriented pyrolytic graphite (HOPG, 10 × 10 mm^2^, Optigraph^©^) crystal is used inside a three-electrode electrochemical cell. The graphite is exfoliated by an adhesive tape along an edge of the sample. The 2 M H_3_PO_4_ solution has been purified by bubbling Ar gas (5.0 grade pure) inside a separator funnel for several days. A Pt wire is used both as counter electrode (CE) and reference electrode (RE). Further information on Pt electrodes is reported in [7].

After the electrochemical treatment, the sample is dried under pure nitrogen (N_2_ 5.5 grade) for several seconds. The nitrogen flux is directed perpendicularly to the sample surface. The N_2_ vessel outlet is set to 0.25 bar above the atmospheric pressure. Following this procedure, we always recognize three morphological regions (labeled as A, B and C) on the HOPG surface by optical microscopy.

### Optical microscopy analysis

An Olympus^©^ BX41 optical microscope is used to characterize different areas of the sample after the intercalation process. The sample was removed from the EC cell, dried with nitrogen and placed below the microscope.

#### Electrochemical atomic force microscopy (EC-AFM)

A commercial (Keysight^©^ 5500 apparatus) AFM is used in these experiments in air and in the electrochemical cell (EC-AFM). The EC-cell is placed on the WE, where a Viton O-ring ensures the seal of the acid solution. The EC-cell and the AFM can be placed inside a protected Ar environment to avoid a progressive degradation of the solution. AFM images are collected in contact mode.

#### Raman spectroscopy

Raman spectra were recorded with a Jobin Yvon Labram HR800 Raman spectrometer coupled with an Olympus BX41 microscope. Spectra were acquired in backscattering geometry using a 50× objective with different excitation lines (457.9 nm Ar^+^ laser, 632.8 nm He–Ne laser, 784.5 nm diode laser).
